# Rescue of the MERTK phagocytic defect in a human iPSC disease model using translational read-through inducing drugs

**DOI:** 10.1038/s41598-017-00142-7

**Published:** 2017-03-03

**Authors:** Conor M. Ramsden, Britta Nommiste, Amelia R. Lane, Amanda-Jayne F. Carr, Michael B. Powner, Matthew J. K. Smart, Li Li Chen, Manickam N. Muthiah, Andrew R. Webster, Anthony T. Moore, Michael E. Cheetham, Lyndon da Cruz, Peter J. Coffey

**Affiliations:** 1 Department of Ocular Biology and Therapeutics (ORBIT), Institute of Ophthalmology, University College London, 11–43 Bath Street, London, EC1V 9EL UK; 2 NIHR Biomedical Research Centre at Moorfields Eye Hospital NHS Foundation Trust and UCL Institute of Ophthalmology, London, EC1V 2PD UK; 3 Moorfields Eye Hospital NHS Foundation Trust, 162 City Road, London, EC1V 2PD UK; 4 Division of Optometry and Visual Sciences, City University London, Northampton Square, London, EC1V 0HB UK; 5 UCSF School of Medicine, Koret Vision Center, 10 Koret Way, San Francisco, CA 94143 USA; 6 Center for Stem Cell Biology and Engineering, NRI, UC Santa Barbara, USA

## Abstract

Inherited retinal dystrophies are an important cause of blindness, for which currently there are no effective treatments. In order to study this heterogeneous group of diseases, adequate disease models are required in order to better understand pathology and to test potential therapies. Induced pluripotent stem cells offer a new way to recapitulate patient specific diseases *in vitro*, providing an almost limitless amount of material to study. We used fibroblast-derived induced pluripotent stem cells to generate retinal pigment epithelium (RPE) from an individual suffering from retinitis pigmentosa associated with biallelic variants in *MERTK*. MERTK has an essential role in phagocytosis, one of the major functions of the RPE. The MERTK deficiency in this individual results from a nonsense variant and so the MERTK-RPE cells were subsequently treated with two translational readthrough inducing drugs (G418 & PTC124) to investigate potential restoration of expression of the affected gene and production of a full-length protein. The data show that PTC124 was able to reinstate phagocytosis of labeled photoreceptor outer segments at a reduced, but significant level. These findings represent a confirmation of the usefulness of iPSC derived disease specific models in investigating the pathogenesis and screening potential treatments for these rare blinding disorders.

## Introduction

Inherited retinal dystrophies represent a heterogeneous group of diseases, which are now the most common cause of registered blindness in the working age population in the UK^1^. Few naturally occurring disease models exist for this group of disorders. Furthermore, access to affected retina and retinal pigment epithelium (RPE) is restricted, providing a very limited amount of human tissue to study. With the advent of induced pluripotent stem cell (iPSC) technology^2^ disease specific human models can be generated *in vitro* with a theoretically limitless supply of cells that can be differentiated to the tissue of interest to study these diseases and test drugs or genetic therapies^3, 4^.

The RPE is a cellular monolayer that has an essential supporting role to the photoreceptors in the retina, being involved in retinol cycling, nutrient transport, growth factor production and phagocytosis of the discarded photoreceptor outer segments^5^.

MER receptor tyrosine kinase (MERTK) is a tyrosine kinase that is expressed at the apical surface of the RPE. It is a key regulator of the recognition and internalisation of photoreceptor outer segments (POS) during the process of phagocytosis by RPE cells^6^. The MERTK protein is a 999 amino acid polypeptide with a molecular weight of 110 kDa, which is subject to post-translational modifications such as N-linked glycosylation^7^. MERTK deficiency results in a form of retinitis pigmentosa, RP38, a rod cone dystrophy for which there is no effective treatment^8^.

Recently, the results of a gene therapy trial with AAV2 mediated delivery of *MERTK* to RP38 individuals have been published, with mixed results^9^. This highlights the requirement for new strategies to test potential therapeutics for these genetic disorders. One potential method is to target a specific variant using small molecules. In the case of RP38, several of the known human *MERTK* variants result in a premature stop codon^10–14^. In these cases, a potential strategy is to employ translational readthrough inducing drugs (TRIDs), which stimulate the bypass of the premature stop codon^15^. This allows continuation of translation, restoring a full-length protein. TRIDs have been shown to be effective in restoring protein and function in previous studies of iPSC-RPE from an individual with X-linked retinitis pigmentosa caused by a mutation in RP2^16^.

In this study, we have generated fibroblasts cells from a skin biopsy of an individual with compound heterozygous variants in the *MERTK* gene. These cells were then reprogrammed to iPSC and differentiated into mature RPE cells with an aim to characterise this iPSC derived model of MERTK retinitis pigmentosa, and employ TRIDs to restore MERTK function.

## Results

### Characterisation of MERTK variant clinical phenotype and iPSC derived RPE

**Figure 1:**
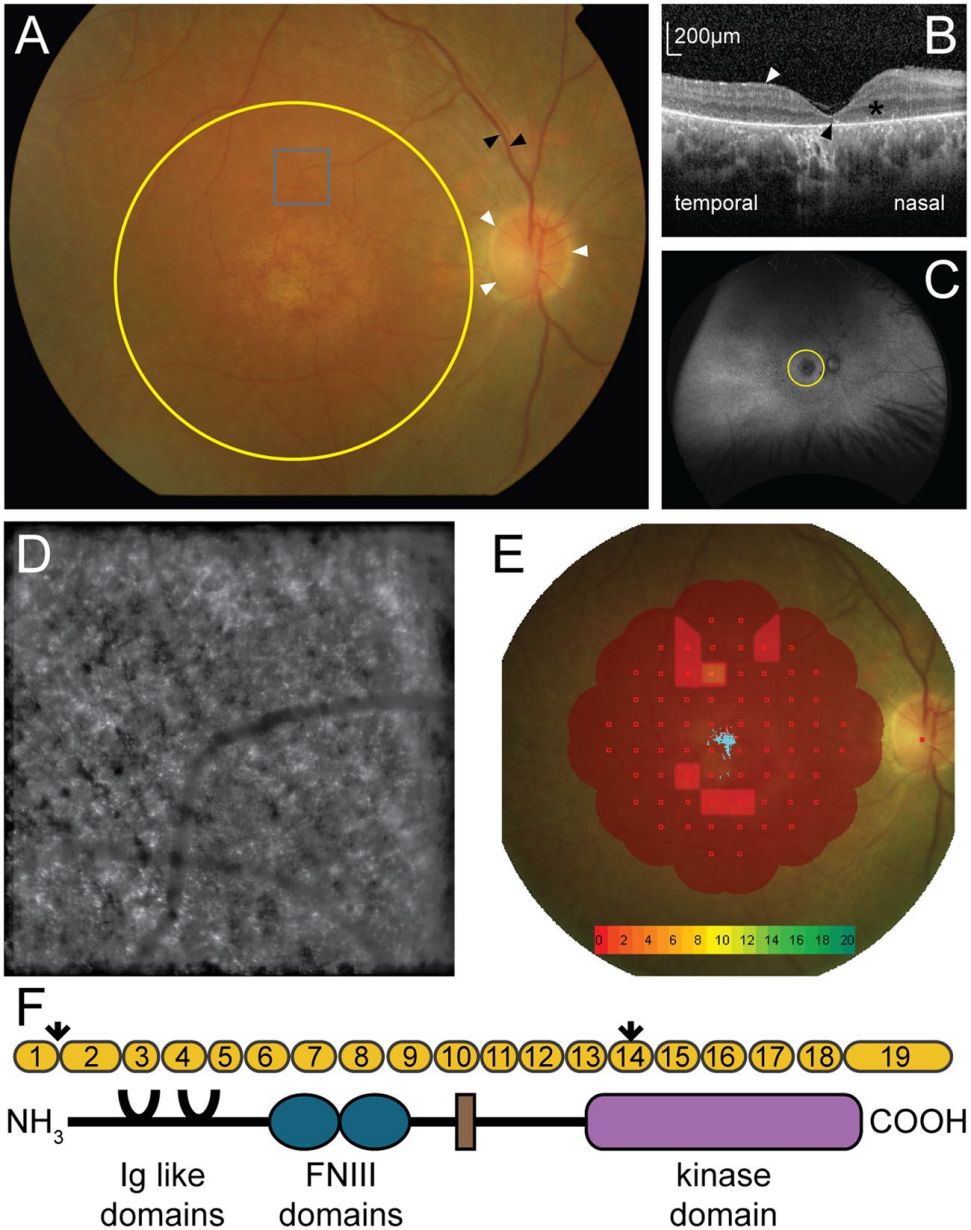
Phenotypic characterisation of an individual with MERTK associated retinal dystrophy. (**A)** Fundus photo of the right eye showing the optic nerve (white arrows), retinal vessels (black arrows) and macula area (yellow circle). (**B)** Optical coherence tomography at the right fovea showing epiretinal fibrosis (white arrow), the outernuclear layer (*) and expected position of the ellipsoid layer (black arrow). (**C)** Widefield Optos autofluorescence image of the right retina showing the macula (yellow circle) and peripheral retina. (**D)** ImagineEyes adaptive optics image of the right retina at 5 degrees superior to the fovea (grey box on panel A). (**E)** Interpolated map of Nidek microperimetry of the right macula, with decibel scale (collection of blue points in centre of macula = fixation). (**F)** Schematic of *MERTK* gene displaying the exons (yellow), the two variants (arrows), one in the first base of intron 1, predicted to interfere with splicing and a premature stop in exon 14, and the protein domains of MERTK.

We obtained a skin biopsy from a 26-year-old male (GC15566) with compound heterozygous variants in the *MERTK* gene, consisting of a single base change in the first base of intron 1 (61+1 G>A) and a nonsense variant in exon 14 (1951 C>T)^13^. Snellen visual acuity was 6/36 in the right eye and 3/60 in the left. Fundus examination revealed bilateral central retinal degeneration, consistent with an inherited maculopathy, bilateral optic nerve head pallor and narrowed and atrophic retinal blood vessels (Fig. 1A). There were no areas of hyperpigmentation noted in the peripheral retinal examination. Optical coherence tomography (Spectralis, Heidelberg) shows an increased right macular retinal thickness nasally, a mild degree of epiretinal fibrosis, general preservation of the outernuclear layer but intermittent loss of the ellipsoid zone and RPE layers (Fig. 1B). Blue light, widefield autofluorescence (Optos) shows a hypofluorescence centrally, matching the area of central macular degeneration with general preservation of the periphery apart from a few punched out lesions outside the macular area (Fig. 1C). Adaptive optics assisted flash photographs taken with the Rtx-1 fundus camera (ImagineEyes) of the right eye at 5 degrees superior to the fovea (grey box on Fig. 1A) shows disorganised, scattered reflections consistent with photoreceptor loss (Fig. 1D). Macular microperimetry (Nidek) shows reduced retinal sensitivity at the macula (Fig. 1E), with the blue collection of spots marking the fixation of the eye.

**Figure 2:**
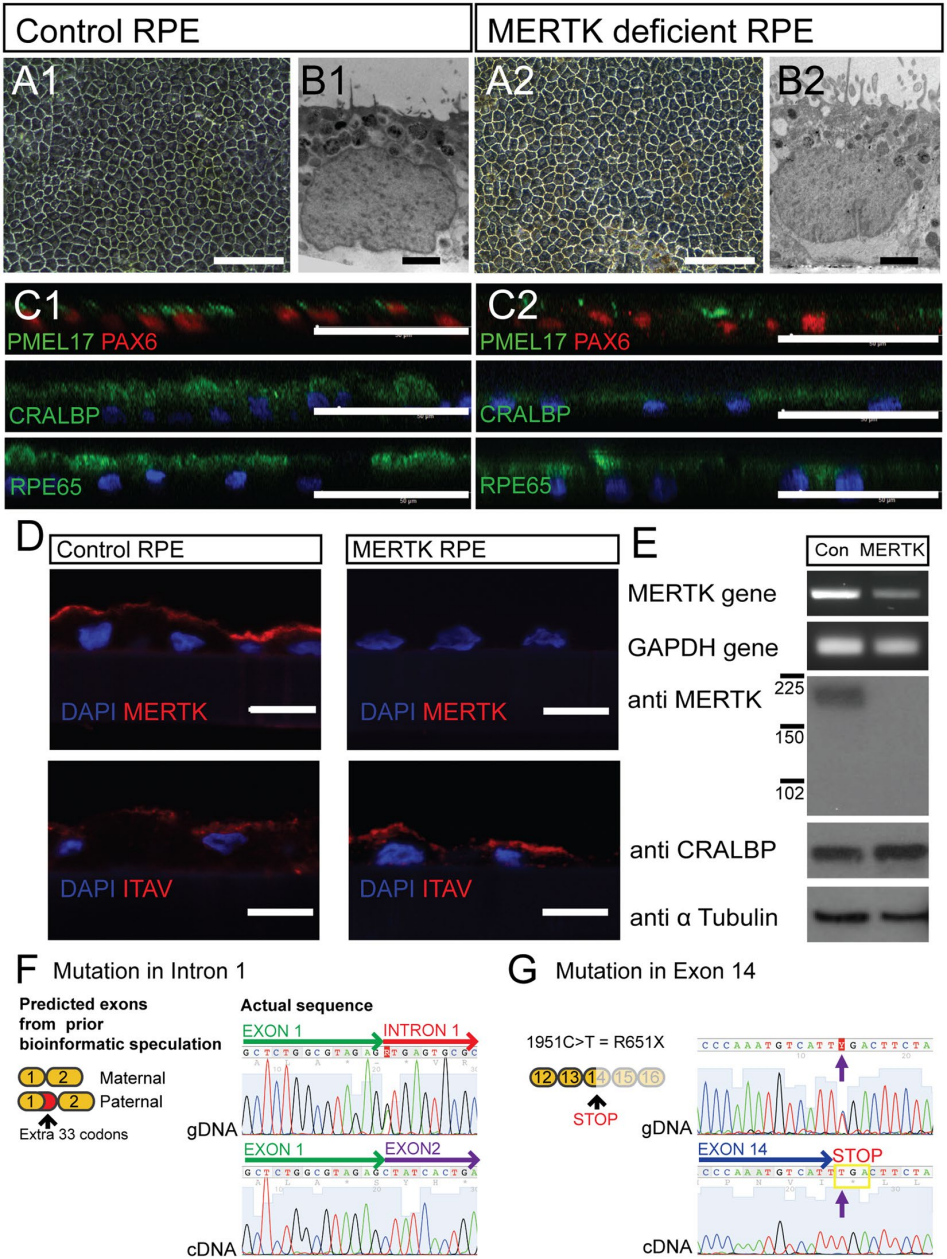
Characterisation of control and MERTK-RPE. (**A1**,**2)** Light micrograph of control and MERTK-RPE cells in culture (scale bar 50 μm). (**B1**,**2)** Electron micrograph of control and MERTK-RPE (scale bar 1 μm). (**C1**,**2)** Immunocytochemistry of iPSC derived RPE (scale bar 40 μm). (**D)** Immunohistochemistry of the control RPE and MERTK-RPE (scale bar = 10 μm). (**E)** RT PCR gel electrophoresis and Western blot of the *MERTK* gene and protein in the control RPE and MERTK-RPE. (**F**) gDNA sequence from MERTK fibroblasts of the exon 1/intron 1 junction of the *MERTK* gene compared to the corresponding MERTK-RPE derived cDNA sequence at the exon 1/exon 2 border. (**G**) gDNA sequence of MERTK fibroblasts compared to MERTK-RPE cDNA sequence in exon 14 of the *MERTK* gene.

Dermal fibroblasts were expanded from the biopsy and reprogrammed into iPSC using episomal vectors in parallel with a control human BJ fibroblast cell line (Figure S1A–C). The iPSC were differentiated into RPE using a spontaneous method of differentiation^17^. Pigmented RPE cells were manually purified and cultured as a monolayer that gradually re-pigmented after 6–8 weeks (Fig. 2).

Control iPSC derived RPE (control RPE) and MERTK deficient iPSC derived RPE (MERTK-RPE) had similar cobblestone morphology and pigmentation (Fig. 2A1,2). Electron microscopy revealed the presence of basal nuclei, apical microvilli, tight junctions and melanosomes in both control and MERTK-RPE (Fig. 2B1,2). Sectioned monolayers of both RPE cells showed highly polarised and mature cells expressing the RPE-associated marker proteins PMEL17, PAX 6, CRALBP and RPE65 (Fig. 2C1,2). Integrin αV was detectable in both control and MERTK-RPE cells, at their apical surface (Fig. 2D).

*MERTK* mRNA was expressed in both the control RPE and MERTK-RPE, but at a lower level in the MERTK-RPE (Fig. 2E). However, the MERTK protein was only detectable in the control RPE, whilst the CRALBP protein, an RPE cell marker was present in both control RPE and MERTK-RPE at equal levels (Fig. 2D,E). Analysis of the *MERTK* gDNA sequence confirmed a change in the first base of intron 1, as previously described^13^. Within the coding sequence of the gDNA five heterozygous variants were detected (Figure S2), two missense, two synonymous and one nonsense. Analysis of the MERTK cDNA prepared from MERTK-RPE cells showed only the wild type sequence at the exon 1/2 border (Fig. 2F). In the *MERTK* cDNA from the MERTK-RPE, only one variant was observed at each of the five positions that had been noted to be heterozygous in the gDNA (Figure S2). Of these five, only one differed from the wild type, NCBI sequence. This was the C>T variant at coding position 1951, leading to a premature stop codon in exon 14 (Fig. 2G). There was no evidence of a truncated MERTK protein by Western blot analysis, suggesting that if a truncated protein was produced it is unstable and degraded (Fig. 2D).

### MERTK iPSC derived RPE are unable to phagocytose

**Figure 3:**
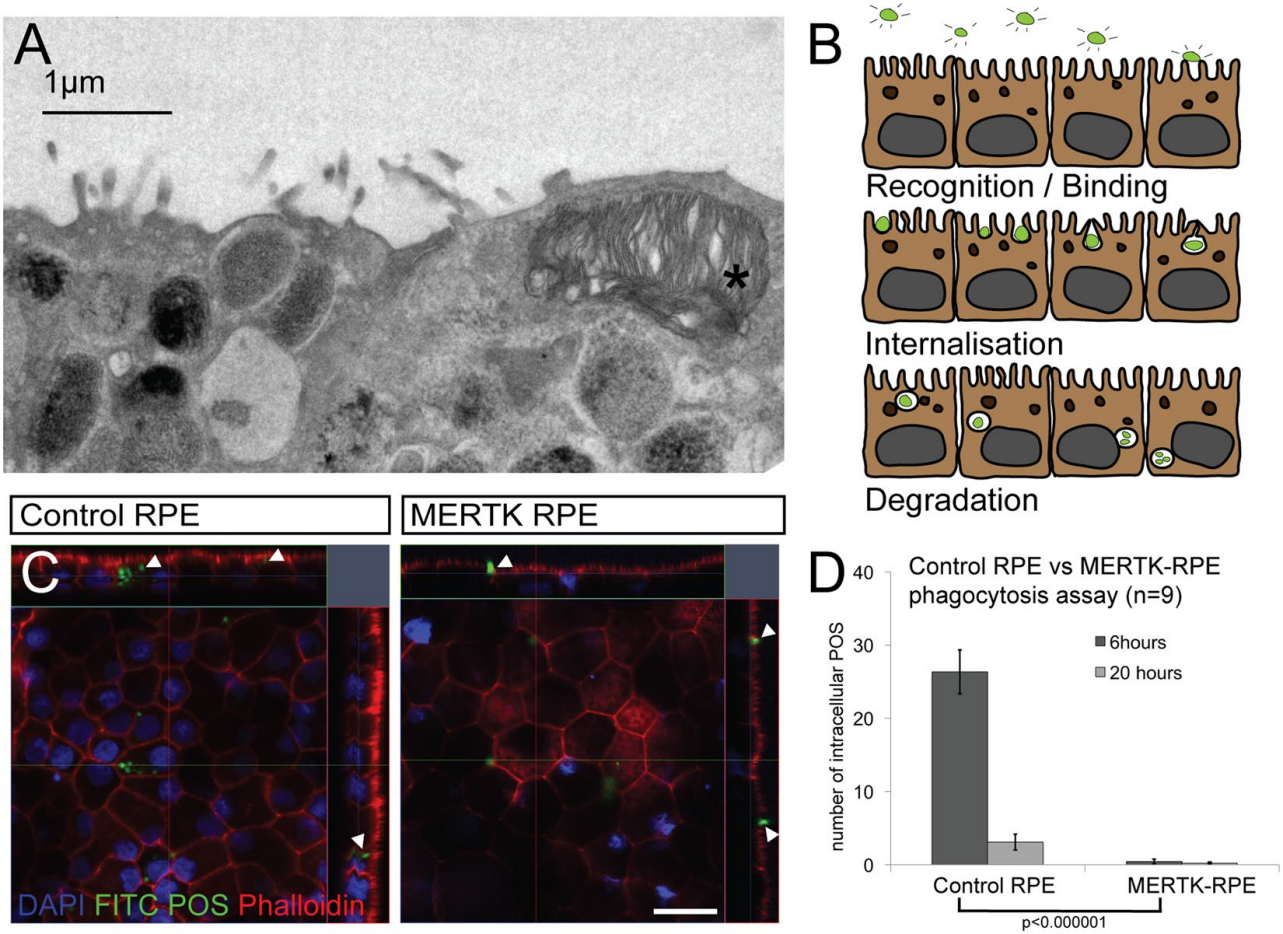
MERTK-RPE are unable to phagocytose. (**A**) Electron micrograph of the apical surface of the control RPE 4 hours following the addition of isolated photoreceptor outersegments (POS) *. (**B)** Schematic of the process of POS phagocytosis by RPE using FITC labeled POS. (**C**) Orthogonal representations of confocal Z stacks of the control RPE and MERTK-RPE 6 hours following the addition of FITC labeled POS (scale bar = 20 μm), arrows highlight POS. (**D**) Graphical representation of phagocytosis between control RPE and MERTK-RPE at 6 and 20 hours (error bars represent the mean ±1SEM and p value derived from students t-test).

The proposed function of MERTK in RPE cells is to mediate the engulfment of POS during phagocytosis^6, 18^. To test this hypothesis in our MERTK-RPE, we modified established phagocytosis assays^19, 20^, exposing the RPE cells to isolated POS *in vitro*. Four hours after the addition of isolated POS to both the control and MERTK-RPE, POS are clearly visible inside the control RPE by electron microscopy (Fig. 3A), with none observed in the MERTK-RPE (data not shown). After 6 hours of POS exposure, FITC labeled POS are clearly visible inside the control RPE but were not observed in the MERTK-RPE on confocal orthogonal stacks (Fig. 3C,D) (26.33 POS internalised/0.01 mm^2^ in control RPE, SEM = 2.99; 0.44 in MERTK-RPE, SEM = 0.34; n = 9; p < 0.000001).

### Translational readthrough inducing drugs are able to restore expression of MERTK in the MERTK-RPE and improve phagocytosis

**Figure 4:**
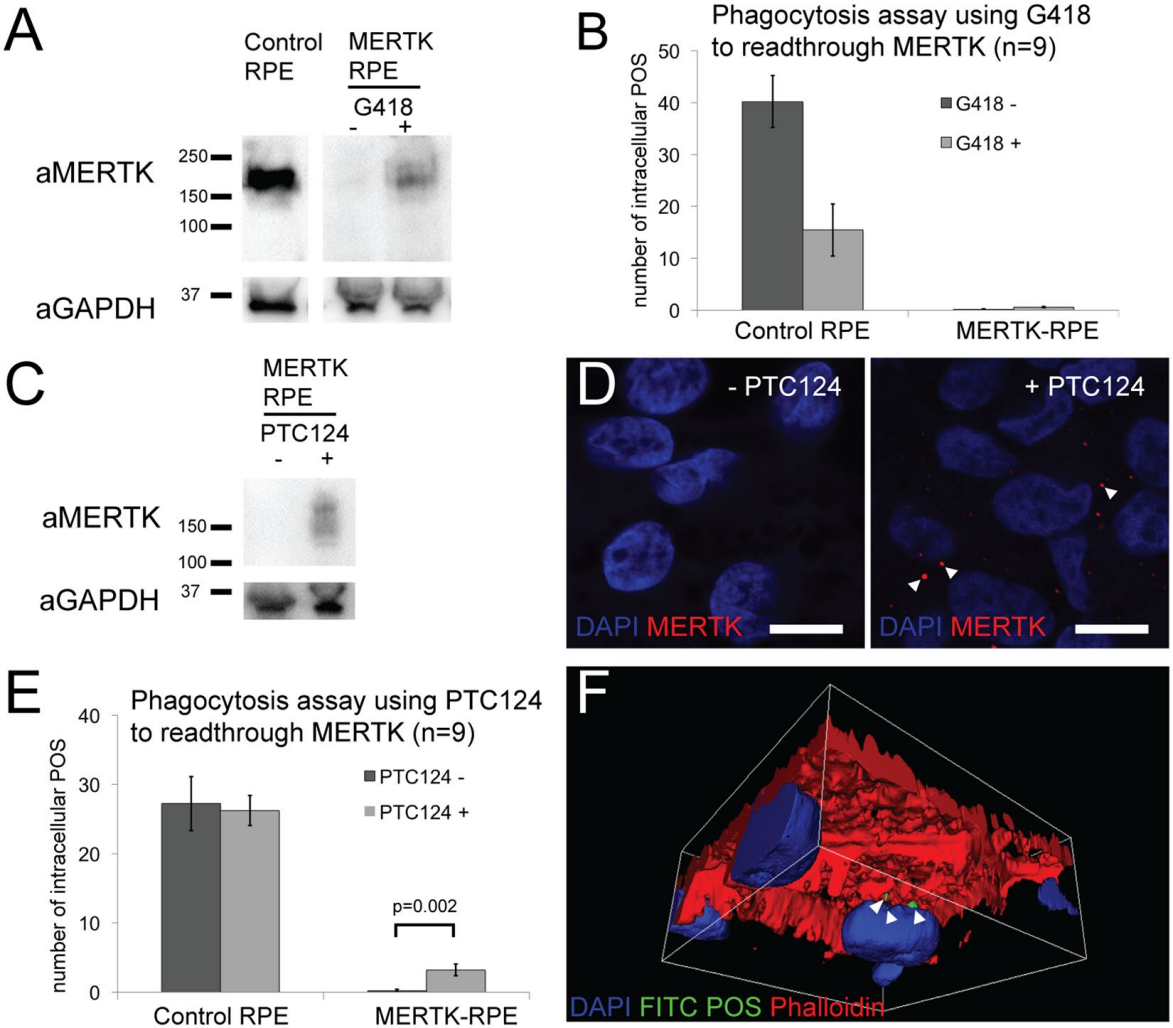
Translational readthrough inducing drugs are able to restore expression of MERTK in the MERTK-RPE and have an effect on phagocytosis. (**A)** Cropped Western blot of the MERTK protein in control RPE and MERTK-RPE treated with G418. (**B**) Outcome of phagocytosis assay with and without G418 in control RPE and MERTK-RPE. (**C**) Cropped Western blot of MERTK protein in control and MERTK-RPE treated with PTC124. (**D**) Immunocytochemistry of MERTK protein in MERTK-RPE treated with PTC124, arrows highlight MERTK expression (scale bar 10 μm). (**E**) Outcome of phagocytosis assay with and without PTC124 in control and MERTK-RPE. (**F**) 3D model derived from confocal stack, viewed from below, showing MERTK-RPE treated with PTC124, 6 hours after the addition of PTC124; arrows show internalised POS (error bars represent the mean ± 1SEM and p values derived from students t-test).

Treatment of the MERTK-RPE cells with 750 μM of the aminoglycoside G418 resulted in the detection of an immunoreactive species with the expected mobility of the MERTK protein. This immunoreactive species was not present in the vehicle treated MERTK-RPE cells by Western blot analysis (Fig. 4A). This rescue of some detectable protein with G418, did not appear to restore phagocytic function in the MERTK-RPE as revealed by the phagocytosis assay (Fig. 4B) (0.11 in untreated, SEM 0.11; 0.55 treated, SEM = 0.33; n = 9; p > 0.1). Furthermore, when control RPE was treated with G418, phagocytosis was reduced compared to the vehicle treated, suggesting G418 may inhibit POS phagocytosis (40.22 in untreated, SEM = 5.02; 15.44 in treated, SEM = 4.46; n = 9; p < 0.001) and this could mask any restoration of MERTK function in the MERTK-RPE.

Treatment with the small molecule PTC124 (10 μgml^−1^), led to the detection of a MERTK immunoreactive species of the correct mobility by Western blot analysis that was not present in vehicle-treated MERTK-RPE cells (Fig. 4C). MERTK immunoreactivity was also detectable on immunocytochemistry in the MERTK-RPE treated with PTC124 (Fig. 4D).

Furthermore, the treatment with 10 μgml^−1^ PTC124 in MERTK-RPE cells stimulated an increase in phagocytic activity (12% of control RPE level) after 6 hours exposure to POS *in vitro* (0.22 in untreated, SEM = 0.22; 3.22 in treated, SEM = 0.85; n = 9; p = 0.002). Importantly, treatment with PTC124 did not seem to have an effect on control RPE phagocytic activity (Fig. 4E,F).

## Discussion

A deeper phenotyping of this individual with variants in *MERTK* reveals that, outside the macula area, there is little hyperpigmentation, a sign of irreversible retinal damage. In addition, there is some preservation of the gross retinal anatomy and persistence of the retinal layers, displayed by OCT. Furthermore, while there is evidence of photoreceptor loss on adaptive optical imaging, there is some preservation of function as outlined by the microperimetry. Not only is there some ability to see a stimulus above the baseline, the individual has good fixation, throughout the test. Further evidence that this represents a less severe case of MERTK-RP comes from comparison with other individuals with variants in MERTK^8–14^. The results of the phenotyping displayed in Fig. 1 are thus encouraging, as any translation of therapy would have its best results in a milder disease form.

From a skin biopsy of this individual with compound heterozygous variants and predicted loss of function in the *MERTK* gene we derived dermal fibroblasts which were reprogrammed to iPSC and then differentiated into RPE cells. These RPE cells have many of the characteristics of primary RPE cells; they grow as a epithelial monolayer with a cobblestone appearance, contain apical pigmented melanosomes, basal nuclei and express proteins involved in the process of retinol cycling^21^. Both the control RPE and the MERTK-RPE express the *MERTK* mRNA as evidenced by RT-PCR but the MERTK-RPE mRNA was present at a lower level. This difference in expression was more pronounced at the protein level with total absence of detectable MERTK protein in the MERTK-RPE.

The gDNA sequence of the *MERTK* gene in the MERTK variant individual revealed five heterozygous variants within the coding sequence. However, at each of these positions in the *MERTK* cDNA from the MERTK-RPE, only one of the variants was detectable. This suggests that only one allele is expressed; this is the allele that includes the premature stop codon in exon 14. We also examined the sequence at the exon 1/2 border as the individual’s *MERTK* genomic sequence suggested that the variant in intron 1 would likely lead to a different splicing pattern and the insertion of an extra 11 codons between exon 1 and exon 2^13^. Our analysis of the *MERTK* cDNA sequence from the MERTK-RPE revealed a sequence at the exon 1/2 border that matches that of the normal *MERTK* transcript (NM_006343.2 NCBI database). These data are consistent with the hypothesis that the allele carrying the variant in the first base of intron 1 is not expressed at the RNA level or is rapidly removed. However, the allele carrying the premature stop codon in exon 14 is detectable. This suggests that in this case, the MERTK defect could be amenable to TRIDs.

Both the control RPE and MERTK-RPE express Integrin αV, a protein required for the rhythmic activation of MERTK^18^, at the apical surface, suggesting that the cells are otherwise primed for phagocytosis. Functional phagocytosis by the control RPE cells was confirmed by electron microscopy following exposure to isolated POS. The POS were clearly engulfed and internalised by the control RPE following a 4 hour exposure to POS. In contrast, no internalised POS were observed in the MERTK-RPE cells. Internalisation of FITC-labeled POS was also observed and quantified in control RPE using confocal stack analysis. In comparison to the control RPE, no internalised POS were observed in MERTK-RPE cells. In addition, it became evident in the control RPE that a 6-hour time point was preferable to a 20-hour assay time point, as at this longer interval, the FITC fluorophore is likely degraded by the RPE cells. A comparison of the number of bound but not internalised POS was measured between the control and MERTK-RPE during phagocytosis (Figure S3). The difference just reached significance by t-test (0.03) and this minor difference could be accounted for by the small contribution of MERTK to binding POS, rather than the major contribution of Integrin αVβ5 in binding POS. This suggests that the major difference noted in phagocytosis between the control and MERTK-RPE occurs at the internalisation phase.

Exposure of the MERTK-RPE to G418, an aminoglycoside related to the antibiotic gentamicin, was able to restore expression of a protein of similar size to MERTK that was not present in the untreated cells. However, this restoration of protein did not correlate into a functional rescue, as the MERTK-RPE were still unable to phagocytose POS. This might be due to a toxic effect of the G418 as it was noted that the control RPE had decreased levels of phagocytosis in the presence of the G418. Aminoglycosides are known to be toxic to the retina^22^, but the exact mechanism is not clear^23^. Interference with normal RPE function could be a potential mechanism. G418, one of the more toxic aminoglycosides, is thought to cause toxicity by binding to mitochondrial ribosomes which are similar to prokaryotic ribosomes. In doing so they inhibit mitochondrial protein synthesis and cause oxidative stress. RPE are known to have high levels of mitochondria in order to provide energy for processes like phagocytosis which may render them particularly sensitive the aminoglycoside toxicity^24^.

Following treatment of the MERTK-RPE with another TRID, PTC124, a small molecule which has been approved by the European Medicines Agency for Duchenne muscular dystrophy^25^, we were able to restore expression of MERTK in the MERTK-RPE. The immunoreactivity was present, however, as multiple bands. One explanation for this is that the protein is differentially post-translationally modified depending on which amino acid is used during the readthrough process. Furthermore, in a phagocytosis assay, the PTC124 treatment restored 12% of the phagocytic function to the MERTK-RPE cells. It is debatable whether an improvement of 12% would be enough *in vivo* to slow the progression of the disease. PTC124 has been used to treat Duchenne muscular dystrophy^26^ and cystic fibrosis^27^. In the preclinical studies of PTC124 for treatment of Duchenne muscular dystrophy (DMD), the drug was able to restore 20% of the protein in a mouse model of the disease and this partially restored protein was shown to localise to the correct cellular compartment in all muscles biopsied. In addition, the treated mice exhibited improved protection from induced injury, an established assay of DMD progression in the model. In phase 1 clinical trials, PTC124 had a very good safety profile and the functional rescue of protein translated, with 34% of subjects taking the drug showing qualitative increase in immunofluorescence for the protein in post treatment muscle biopsies. Furthermore, in phase 2 trials, patients treated with the drug were able to walk further in standardised timed tests than those taking placebo, and while this did not meet significance, other endpoints did, including slowing of disease progression^26^. This level of rescue is comparable to the results shown herein, suggesting that only partial recovery of the protein may facilitate a functional improvement *in vivo*.

The mechanism of action of TRIDs is not completely understood but the drugs are thought to interfere with ribosomal fidelity so that a near-cognate aminoacyl tRNA can bind at the site of a premature stop codon resulting in the incorporation of another amino acid in its place, allowing translation to continue^15^. It is also postulated that the readthrough effect of TRIDs is dependent not just on the nonsense variant but also in the context of the local sequence, the particular bases that make up the stop codon and the stability of the resulting protein. The TRID PTC124 performs best when acting on the UAG stop codon followed by a cytosine^28^, which is the sequence of the *MERTK* premature stop codon presented here and may account for the efficiency observed in this case. We acknowledge that this is the second MERTK iPSC RPE model^29^, however it is the first to show a treatment effect and amelioration of function.

iPSC models offer an attractive alternative to animal models as they allow the study of human tissue. The Royal College of Surgeons (RCS) rat, a natural animal model that harbours a spontaneous homozygous deletion in the *MERTK* gene has been used for decades as a general model of retinal degeneration^30, 31^ and a specific model of MERTK related retinal disease. However, the RCS genotype^32, 33^, does not recapitulate any observed in man^10^ thus diminishing its use as a clinical model in the post genome age.

## Conclusion

We have been able to create and characterise iPSC derived RPE cells with MERTK deficiency to create an accurate *in vitro* model of human disease. Our results show that the MERTK-RPE cells have a defect in the engulfment of POS. Using the translational readthrough inducing drugs G418 and PTC124 we were able to restore expression of the *MERTK* gene to detectable full-length protein in the MERTK deficient RPE. Moreover, the PTC124 compound was able to rescue 12% of the phagocytic function of the RPE cells. It may be possible, therefore, to use TRIDs to treat retinitis pigmentosa due to nonsense variants in *MERTK*, which hitherto has no effective therapy. Furthermore, human iPSC derived models may also be useful to bridge the gap between preclinical animal studies and clinical trials to test other treatments such as gene therapies.

## Materials and Methods

### Clinical phenotyping

Fundus photos were captured with a digital camera and Topcon image capture system. Widefield images and autofluorescence images were captured using the Optos 200Tx retinal imaging system. Optical coherence tomography images and autofluorescence images were captured using a Heidelberg Spectralis. Microperimetry was performed using the Nidek MP-1 microperimeter set to Humphrey 10-2(20 db) with a white 200 ms Goldman III stimulus and a white 2° single cross fixation target. Adaptive optics (AO) images were captured using the Imagine Eyes rtx1™ Adaptive Optics Retinal Camera.

### Generation of fibroblasts from a skin biopsy

The research adhered to the tenants of the Declaration of Helsinki and was approved by the Moorfields Eye Hospital Ethics Committee and the Health Research Authority NRES Committee London. Informed, written consent was gained and a biopsy was obtained from an individual with a MERTK variant and fibroblasts cultivated from it as previously described^16, 34^.

### Reprogramming of fibroblasts

Control fibroblasts (BJ line) were obtained from Stemgent. MERTK and control fibroblasts were reprogrammed to iPSC with episomal delivery of SOX2, OCT3/4, KLF4, L-MYC and LIN28 (27077, 27078, 27080, Addgene) using a protocol that has previously been described^16, 35^.

These stem cells had a normal morphology, expressed markers of pluripotency, had a normal karyotype and were able to differentiate into endoderm, mesoderm and ectoderm (Figure S1D–F).

### Maintenance of iPSC, generation of RPE

iPSC colonies were cultured on Matrigel hESC-qualified matrix (354277, BD Biosciences) in Essential 8 (A1517001, Thermo Fisher). Once 80% confluent, the media was changed to Differentiation Media^17^. From here on, cultures were fed twice weekly for at least a further 8 weeks until pigmented foci were observed. These pigmented foci were isolated manually and purified, fed twice weekly with X-VIVO 10 (LZBE 04-743Q, Lonza) with gentamicin (ThermoFisher). All experiments were performed with passage 2 RPE aged 8 to 16 weeks.

### Phagocytosis assay

Photoreceptor outer segments (POS) were purified from fresh sheep eyes obtained from a local abattoir (Chelmsford, UK) using a previously published protocol^19^. Some POS were labeled with Fluorescein-5-Isothiocyanate (FITC ‘Isomer I’, F1906, ThermoFisher). Samples for electron microscopy analysis of phagocytosis used unlabeled POS. The labeled or unlabeled POS were added to DMEM with 4.5 g/L glucose, L-glutamine and pyruvate (41966029 ThermoFisher), 10 mM GAS6 (885-GS-050, R&D systems, MN, USA) and 10% FBS (10099141, ThermoFisher) and exposed to the iPSC derived RPE at a ratio of 10 POS per cell for 6 hours or 20 hours. At the endpoint, the cells were fixed in 4% PFA at 4 C for 20 mins, permeabilised with 0.3% triton in block solution (1X PBS, BSA 0.5%, Glycine 1%, Sodium Azide 0.1%) and stained with rhodamine phalloidin (R415, ThermoFisher) and Hoechst (33342 ThermoFisher) for 20 mins in block solution and analysed on a Zeiss LSM700 confocal microscope. Z stack confocal analysis was undertaken to measure how many POS were internalised in a 101.5 μm by 101.5 μm area, using the orthogonal view function.

### Translational readthrough inducing drug assay

The media was changed 3 days prior to the assay to media containing no gentamicin. The small molecule TRIDs G418 (A1720, Sigma) or PTC124 (S6003, Selleckchem, Munich, Germany) were added to the X-VIVO 10 at the desired concentration and this media added to the cells at 24, 6 and 2 hours prior to lysis for Western blot or fixing for immunocytochemistry. In the experiments assessing TRID effect on phagocytosis, the G418 or PTC124 was added to the cells in X-VIVO 10, at 28 and 10 hours prior fixing for analysis. At 6 hours prior to fixing, the drugs were added to the POS/DMEM/GAS6/FBS mixture outlined in the phagocytosis assay section.

### Electron microscopy

Samples were prepared using standard Epon embedding methods and viewed on a JEOL 1010 TEM. Images were taken with a Gatan Orius SC100B charge-coupled device camera and analysed with Gatan Digital Micrograph^17^.

### Immunocytochemistry

Samples were fixed in 4% paraformaldehyde and permeabilised with 0.3% triton in block solution (1X PBS, BSA 0.5% (A7906, Sigma), Glycine 1%, Sodium Azide 0.1%) and blocked overnight in block solution. Primary (MERTK, Abcam ab52968, 1:1000; PMEL17, Dako HMB45, 1:1000; PAX6, Covance PRB278P, 1:200; CRALBP, ThermoFisher MA1813, 1:200; RPE65, Millipore MAB5428; ITAV, Abcam 92797, 1:500) and secondary (Alexafluor, ThermoFisher, 1:200) antibodies applied for 1 hour each treated with Hoechst (33342, ThermoFisher) for 30 seconds and mounted in ProLong^®^ Gold Antifade Mountant (P10144, ThermoFisher).

### Immunohistochemistry

All samples were fixed in 4% paraformaldehyde for 20 mins and washed with PBS and prepared with a previously published protocol^17^. Immunohistochemistry of samples on slides carried out in a dark humidified chamber. Slides were stained as for immunocytochemistry.

### Western blot

Samples for Western blotting were lysed prepared using previously published protocol^20^ and separated in a 4–20% Mini-PROTEAN^®^ TGX^TM^ Gel (4561093, Bio-Rad, UK). The separated protein in the gel was transferred using the Trans-Blot^®^ Turbo^TM^ Transfer system (1704155, Bio-Rad, UK). The membrane was blocked overnight and then incubated with primary antibody for 1 hour (MERTK, Abcam 52968, 1:1000; GAPDH, Everest EB07069, 1:2000; CRALBP, ThermoFisher MA1813, 1:1000), washed and incubated with horseradish peroxidase-conjugated secondary antibodies (P0448, P0449, Dako, UK). The membrane was incubated in Amersham ECL prime (GERPN2232, Sigma) and images collected on the ChemiDoc™ MP System (1708280, Bio-Rad, UK).

### Genomic DNA extraction

Genomic DNA was extracted from the fibroblasts using the GeneElute™ Mammalian Genomic DNA mini prep kit (G1N10, Sigma).

### Sequencing

Samples for sequencing were amplified using Advantage^®^ 2 PCR Kit (639206, Clonetech) in a Proflex^TM^ PCR machine (4484077, ThermoFisher). PCR products were separated on an agarose gel, and bands extracted and purified using the QIAquick Gel Extraction Kit (28704, Qiagen). Cycle sequencing was performed using the BigDye Terminator v3.1 sequencing kit (4337455, ThermoFisher). Extension products were purified using EDTA/NaOAc/EtOH precipitation. Sample electrophoresis was performed using a 3730 DNA Analyser (ThermoFisher). Sequences were evaluated in MacVector (NC, USA) and compared to NCBI *MERTK* genomic (NG_011607.1) and mRNA (NM_006343.2) sequences.

### RT PCR

RNA was extracted using TRIzol® Reagent (15596026, ThermoFisher) Reverse transcription was performed with the SuperScript® III First-Strand Synthesis System for RT-PCR (18080-051 ThermoFisher). Polymerase chain reactions (PCR) were performed using the Advantage^®^ 2 PCR Kit (639206, Clonetech) with 50 μl reaction volume in a ProflexTM PCR machine (4484077, ThermoFisher).

### Statistics

All statistics are derived from the student t-test.

## Electronic supplementary material

Supplementary Information
