# Supplementary material for: Rescue of the MERTK phagocytic defect in a human iPSC disease model using translational read-through inducing drugs
Source: Sci Rep. 2017 Mar 3;7:51. doi: 10.1038/s41598-017-00142-7 (PMC5427915; doi:10.1038/s41598-017-00142-7)
Supplement: Supplementary file 1 — Supplementary Information [file 41598_2017_142_MOESM1_ESM.pdf]

Rescue of the MERTK phagocytic defect in a human iPSC disease model using translational read-through inducing drugs

Conor M. Ramsden[1,2,3], Britta Nommiste[1], Amelia Lane[1], Amanda-Jayne F. Carr[1], Michael B. Powner[1,4], Matthew Smart[1], Li Li Chen[1], Manickam N. Muthiah[2,3], Andrew R. Webster[1,2], Anthony T. Moore[1,2,5], Michael E. Cheetham[1], Lyndon da Cruz[1,2,3], Peter J. Coffey[1,2,6,*]

[1] Department of Ocular Biology and Therapeutics (ORBIT), Institute of Ophthalmology, University College London, 11–43 Bath Street, London, EC1V 9EL, UK.

[2] NIHR Biomedical Research Centre at Moorfields Eye Hospital NHS Foundation Trust and UCL Institute of Ophthalmology, London, EC1V 2PD, UK.

[3] Moorfields Eye Hospital NHS Foundation Trust, 162 City Road, London, EC1V 2PD, UK.

[4] Division of Optometry and Visual Sciences, City, University of London, Northampton Square, London, EC1V 0HB, UK.

[5] UCSF School of Medicine, Koret Vision Center, 10 Koret Way, San Francisco, CA 94143, USA.

[6] Center for Stem Cell Biology and Engineering, NRI, UC Santa Barbara, USA.

* Corresponding Author email p.coffey@ucl.ac.uk

tel: +44 20 7608 4039

fax: +44 20 7608 6929

Figure S1. Generation and characterisation of iPSC. A Schematic of a skin biopsy and biopsy preparation in a 10cm dish. B iPSC colonies developing from a fibroblast culture. C Purified iPSC colony. D Immunocytochemistry of a stem cell colony showing expression of typical stem cell markers. E Normal karyotype. F Taqman® scorecard box plot showing upregulation of endoderm, mesoderm and ectoderm markers following differentiation.

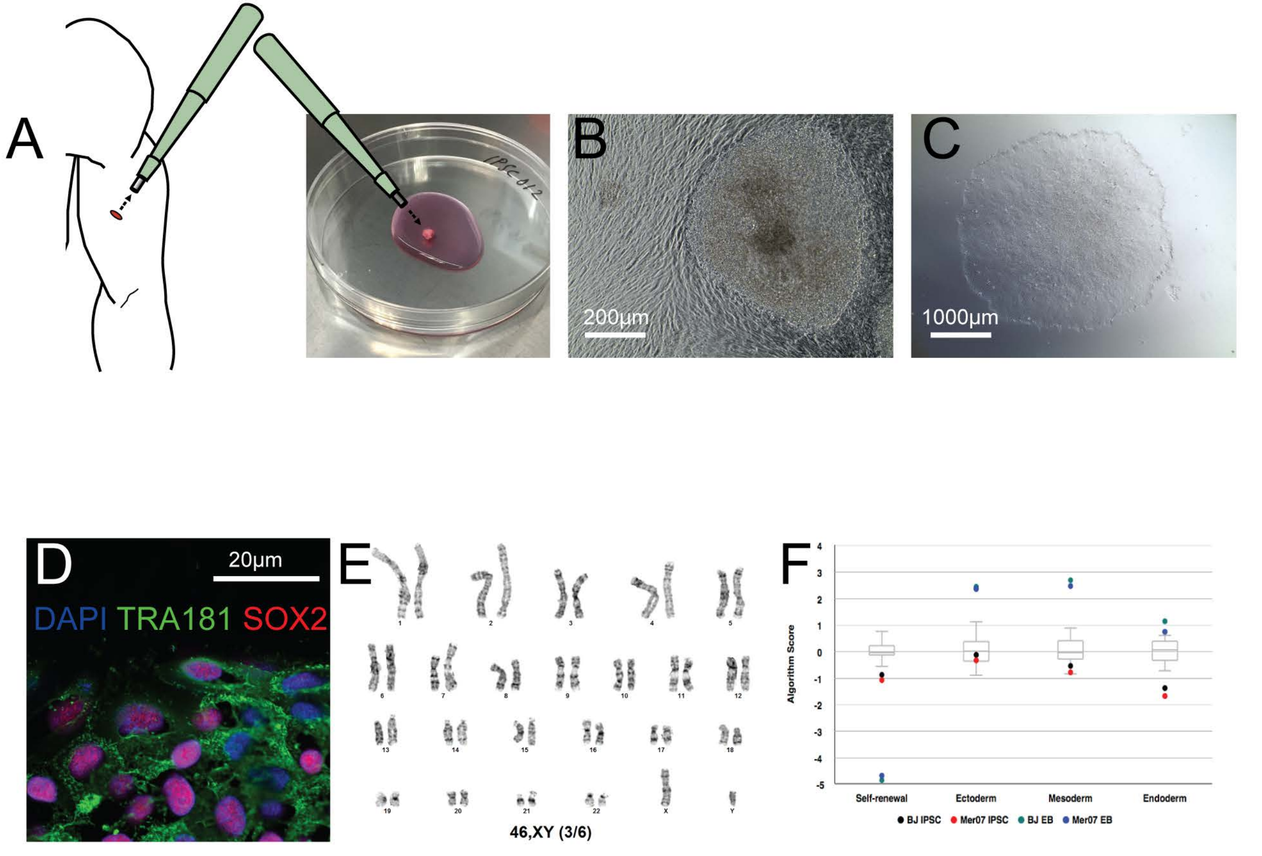

Figure S2. Full length Western blots from Figure 4. In addition to the predicted band for MERTK at approx. 160kDa in all conditions there is a non specific band at 40kDa.

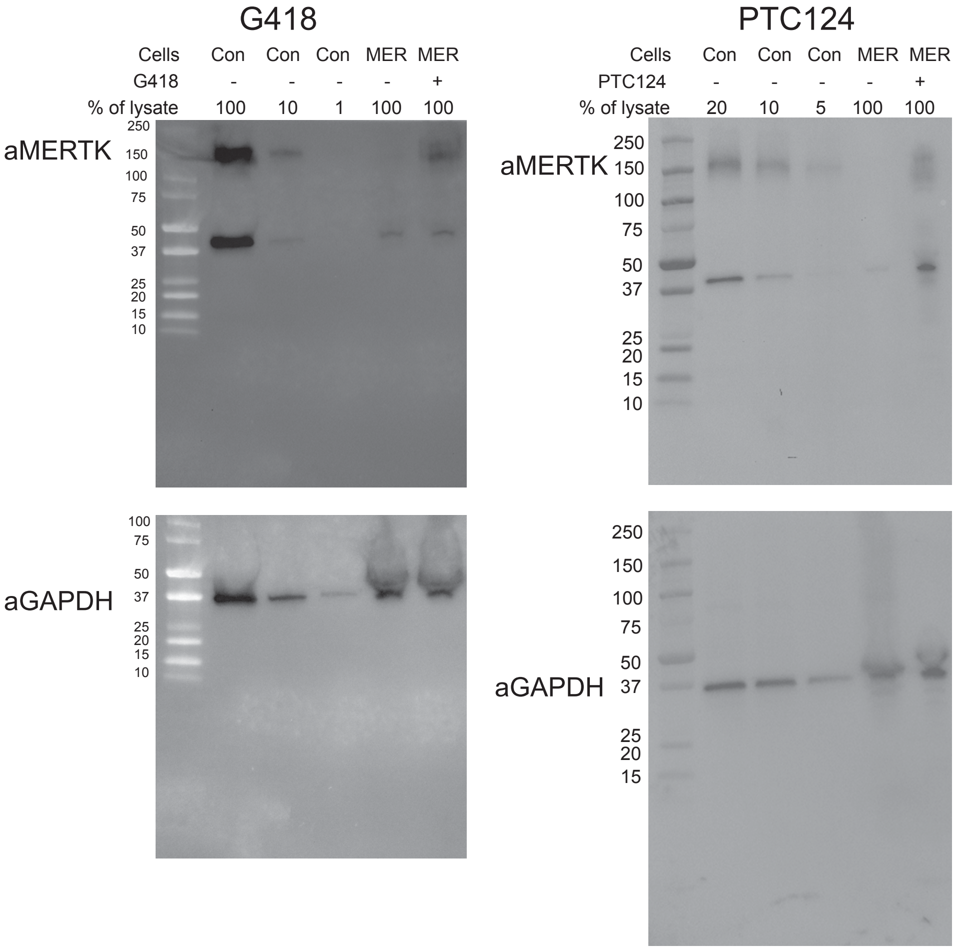

Figure S3. Graphical representation of phagocytosis between control RPE and MERTK-RPE at 6 and 20 hours (error bars represent the mean ±1SEM and p value derived from students t-test).

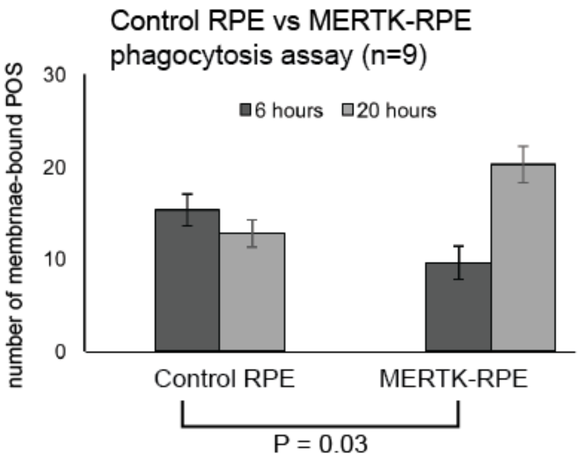

Table S1. Details of sequence variants in the *MERTK* gDNA and cDNA.

Exon	coding position	NCBI reference	*MERTK* gDNA	*MERTK*-RPE cDNA	Predicted effect	rs number	ExAC allele freq
9	1397	G	AG het	G	Missense R466K	rs7604639	0.5972
10	1494	C	CT het	C	Synonymous	rs3811634	0.2385
10	1552	A	AG het	A	Missense I518V	rs2230515	0.5973
14	1881	A	AG het	A	Synonymous	rs1131244	0.5825
14	1951	C	CT het	T	Nonsense R651X	rs119489105	4.95E-05

Table S2. Details of primers used and PCR parameters

***MERTK* gDNA sequencing primers**	**Forward**	**Reverse**
Exon 9	GCCCAGACCTCAGTGTTTTCATTT	CCCAGGTTACTTTCTGGCAATCTG
Exon 10	GATCTCTTCGCATGGTCTCAGCTT	CTTGTCAATACCAGTGGGCAAACA
Exon 14	ACTAGCCCCTGACAACCACTCATC	TTTTTCCTTTGGCACAGAGCAGAT
***MERTK* cDNA sequencing primers**		
Exon 9 & 10	ACACCTCTGCCTTACCACATCTGTAC	CTCCTGGACTCTTTTTCTGATGGCC
Exon 14	GCTCATCATCTTTGGCTGCTTTTGTG	ATATCCACCATGAACTTCAATAGTG
**RT-PCR primers**		
MERTK (exons 1-4)	GGCGACAGGACAGGTTCG	ACTTCGATGTAGATGGGATCAGAC
GAPDH	ACAGTCAGCCGCATCTTCTT	CCCAATACGACCAAATCCGTTG

step 1: 95C for 1 min, step 2: 25 cycles of 95C for 30 sec, *C for 30 sec and 68C for 1 min step 3: 68C for 1 min, where * is the melting temperature of the primer.

Supplementary methods

Karyotype

Karyotyping was performed by The Doctors Laboratory 76 Wimpole Street, London, W1G 9RT, UK, using standard methods.

Scorecard

Pluripotency potential was measured using TaqMan® hPSC Scorecard™ Assay (A15876, ThermoFisher). Stem cell cultures were split: half frozen in TRIzol (15596, ThermoFisher) after 4 days post passage and half used to make embryoid bodies after 6 days. The embryoid bodies were grown for a further 7 days before harvest (following ThermoFisher publication MAN0008384). After TRIzol RNA extraction, first strand synthesis was performed with the High Capacity cDNA RT Kit with RNaseInhibitor (4374966, ThermoFisher) and qPCR performed in the using TaqMan® hPSC Scorecard™ Panel 96w FAST (A15876, ThermoFisher) using TaqMan® Fast Advanced Master Mix (4444557, ThermoFisher). Scorecard analysis was performed using ThermoFisher's cloud based software.
